# A Phosphoinositide 3-Kinase/Phospholipase Cgamma1 Pathway Regulates Fibroblast Growth Factor-Induced Capillary Tube Formation

**DOI:** 10.1371/journal.pone.0008285

**Published:** 2009-12-14

**Authors:** Tania Maffucci, Claudio Raimondi, Shadi Abu-Hayyeh, Veronica Dominguez, Gianluca Sala, Ian Zachary, Marco Falasca

**Affiliations:** 1 Queen Mary University of London, Barts and The London School of Medicine and Dentistry, Blizard Institute of Cell and Molecular Science, Centre for Diabetes, Inositide Signalling Group, London, United Kingdom; 2 Department of Medicine, British Heart Foundation Laboratories, Centre for Cardiovascular Biology and Medicine, University College London, London, United Kingdom; University of Birmingham, United Kingdom

## Abstract

**Background:**

The fibroblast growth factors (FGFs) are key regulators of embryonic development, tissue homeostasis and tumour angiogenesis. Binding of FGFs to their receptor(s) results in activation of several intracellular signalling cascades including phosphoinositide 3-kinase (PI3K) and phospholipase C (PLC)γ1. Here we investigated the basic FGF (FGF-2)-mediated activation of these enzymes in human umbilical vein endothelial cells (HUVECs) and defined their role in FGF-2-dependent cellular functions.

**Methodology/Principal Findings:**

We show that FGF-2 activates PLCγ1 in HUVECs measured by analysis of total inositol phosphates production upon metabolic labelling of cells and intracellular calcium increase. We further demonstrate that FGF-2 activates PI3K, assessed by analysing accumulation of its lipid product phosphatidylinositol-3,4,5-P_3_ using TLC and confocal microscopy analysis. PI3K activity is required for FGF-2-induced PLCγ1 activation and the PI3K/PLCγ1 pathway is involved in FGF-2-dependent cell migration, determined using Transwell assay, and in FGF-2-induced capillary tube formation (tubulogenesis assays *in vitro*). Finally we show that PI3K-dependent PLCγ1 activation regulates FGF-2-mediated phosphorylation of Akt at its residue Ser473, determined by Western blotting analysis. This occurs through protein kinase C (PKC)α activation since dowregulation of PKCα expression using specific siRNA or blockade of its activity using chemical inhibition affects the FGF-2-dependent Ser473 Akt phosphorylation. Furthermore inhibition of PKCα blocks FGF-2-dependent cell migration.

**Conclusion/Significance:**

These data elucidate the role of PLCγ1 in FGF-2 signalling in HUVECs demonstrating its key role in FGF-2-dependent tubulogenesis. Furthermore these data unveil a novel role for PLCγ1 as a mediator of PI3K-dependent Akt activation and as a novel key regulator of different Akt-dependent processes.

## Introduction

Angiogenesis, the formation of new vessels from existing vasculature, is required for different physiological processes such as development, reproduction, wound healing, and tissue regeneration [1]. On the other hand angiogenesis is involved in pathologies such as arteriosclerosis, diabetic retinopathy, rheumatoid arthritis and tumour growth [2], [3]. Angiogenesis consists of a multistep process involving capillary endothelial cell (EC) migration and proliferation and the formation of three-dimensional structures capable of carrying blood [1]. A complex network of growth factors and cytokines regulates angiogenesis including the family of vascular endothelial growth factor (VEGFs) and fibroblast growth factor (FGFs) [4]. The signalling pathways activated by VEGFs have been extensively investigated in physiological and pathological angiogenesis. For instance the key role of VEGFs in tumour angiogenesis is well recognised [5], [6] and several anti-angiogenic agents targeting VEGFs signalling are now available for anti-cancer strategy. The FGFs family consists of at least 22 factors that play a crucial role in morphogenesis during embryo development and in control of the nervous system, tissue repair and wound healing in the adult organism [7]. On the other hand, several lines of evidence indicate that FGFs, in particular FGF-2, play a key role in tumour angiogenesis [8]. For instance FGF-2 is frequently highly expressed in highly vascularized and advanced cancers [9]–[11] and it has been reported that a synergistic action of FGF-2 and platelet derived growth factor-BB promotes tumour angiogenesis and pulmonary metastasis in mice [8], [12]. In addition FGF-2 has been shown to be associated with resistance to anti-VEGF agents [13]. Germline mutations in FGF receptors have been detected in different cancer types [14] and data suggest that inhibitions of these receptors through small molecule inhibitors or antibodies may be of clinical value [14].

Binding of VEGFs and FGFs to their respective receptors leads to receptor phosphorylation and subsequent activation of signalling proteins including the Ras pathway, the Src family tyrosine kinases, phosphoinositide 3-kinase (PI3K) and phospholipase C (PLC)γ1.

PI3Ks are a conserved family of lipid kinases that catalyze the phosphorylation of the D3 position of the inositol ring of phosphoinositides at the plasma membrane or in specific cellular membrane compartments. Interaction between the newly generated 3-phosphorylated phosphoinositides and distinct structural motifs, such as pleckstrin homology (PH) domain, can regulate the activity of different proteins through their membrane targeting or direct modulation of their enzymatic activity [15]. Among such proteins the serine/threonine kinase protein kinase B (PKB)/Akt, an enzyme involved in diverse intracellular events including cell proliferation, migration, survival and cell cycle progression, is undoubtedly the most studied and best characterised. Several lines of evidence indicate that PI3K plays an important role in VEGF signalling, angiogenesis and regulation of VEGF expression [16]. While the VEGF-dependent activation of PI3K has been extensively studied, the mechanism of FGF-mediated activation of PI3K has been controversial until few years ago when it was demonstrated that a growth factor-induced coordinated assembly of a multi-docking protein complex is necessary for the FGF-2-mediated activation of PI3K [17].

PLCγ1 hydrolyzes phosphatidylinositol-4,5-bisphosphate to generate the second messengers inositol-1,4,5-trisphosphate and diacylglycerol which in turn can activate several downstream effectors including specific protein kinase C (PKC) isoforms leading to many cellular responses [18]. Several lines of evidence indicate that PLCγ1 plays a critical role in angiogenesis, especially in VEGF signalling with data suggesting a role for this enzyme in mediating VEGF-induced cell proliferation and others revealing a role for PLCγ1 in stimulating VEGF-induced differentiation and tubulogenesis [19]–[21]. PLCγ1 deletion results in reduced vasculogenesis and erythropoiesis in mice [22] and in defects in artery formation in transgenic zebrafish models [23]. The role of PLCγ1 in FGF signalling has been less investigated. Although the general mechanism of PLCγ1 activation involves its receptor-mediated tyrosine phosphorylation, we demonstrated that PLCγ1 can also be activated downstream to PI3K in a mechanism involving the interaction between its PH domain and the PI3K product phosphatidylinositol-3,4,5-trisphosphate (PtdIns-3,4,5-P_3_) [24].

Here we show that FGF-2 activates PLCγ1 in human umbilical vein endothelial cells (HUVECs) and that this enzyme is critical for the FGF-2-dependent cell migration and capillary tube formation (angiogenesis *in vitro*). Moreover our data demonstrate that a PI3K-dependent PLCγ1 activation regulates FGF-2-mediated phosphorylation of Akt through PKCα activation, unveiling a novel role for PLCγ1 as a mediator of the PI3K-dependent Akt activation and as a key regulator of different Akt-dependent processes.

## Materials and Methods

### Materials

FGF-2 and VEGF were purchased from Peprotech, siGENOME smartpool for PLCγ1, PLCγ2 and PKCα from Dharmacon; scrambled siRNA from Ambion; U73122, U73343, LY294002, Gö6976, PD98059 and U0126 from Calbiochem; SH5 from Alexis Biochemicals; wortmannin from Sigma. Alternative siRNA targeting PLCγ2 was purchased from Qiagen. Anti PtdIns-3,4,5-P_3_ was purchased from Echelon, anti PLCγ1, anti Akt, anti actin, anti PLCγ2, anti ERK2, anti PKCα from Santa Cruz Biotechnology; anti pSer473 Akt from Cell Signaling Technology.

### Cell lines and cultures

HUVEC were purchased from TCS CellWorks and grown in EGM kit (EBM+singlequots, Lonza) supplemented with 10% FBS. For expression of GFP-PH PLCγ1, HUVEC were electroporated as described [25]. Briefly, cells detached from a 10-cm cell culture dish were resuspended in 400 µl of electroporation buffer (20 mM Hepes pH 7.5, 137 mM NaCl, 5 mM KCl, 0.7 mM Na_2_HPO_4_, 6 mM D-glucose) and incubated with 25 µg DNA in a cuvette for 10 min at room temperature. Cells were electroporated (960 µF, 200 V) by using a Bio-Rad Gene Pulser instrument (time constant ∼26 sec) and then left in the cuvette for a further 10 min at room temperature before plating.

For siRNA-based experiments, confluent HUVEC plated in 6 well plates were transfected by incubating in OPTIMEM containing oligofectAMINE (Invitrogen) and 200 nM siRNA. After 24 h cells were serum starved overnight in M199+0.5% FBS before being used for experiments. Alternatively, number of cells was determined 24 h or 48 h after transfection by manual counting.

### Inositol phosphates production

Cells were labelled with [^3^H]*myo* inositol (PerkinElmer) in inositol free medium M199 supplemented with 2% FBS. After 24 h, cells were incubated in M199 containing 10 mM Hepes and 20 mM LiCl for 15 min before stimulation, then lysed in 0.1 M HCOOH. Total inositol phosphates were separated by anion exchange chromatography by using AG 1-X8 resin (formate form, 200–400 mesh, BioRad) and the total amount assessed by liquid radioactivity counting.

### PI3K activation in vivo

PtdIns-3,4,5-P_3_ production was assessed by thin layer chromatography (TLC) analysis as described [26]. Briefly, cells grown in a 6 well plate were incubated with 0.8 mCi/well [^32^P]phosphoric acid (PerkinElmer) in phosphate free medium M3786 (Sigma) for 3 h before stimulation as indicated. Phospholipids were extracted, dried under nitrogen, resuspended in 100 µl chloroform∶methanol (3∶2) and spotted onto silica plates pre-soaked in water∶methanol (3∶2) containing 1.2% (w/v) oxalic acid and oven baked for 15 min at 110°C. TLC plates were developed in chloroform: acetone: methanol: acetic acid: water (90∶36∶30∶27∶18).

### Migration and Tubulogenesis assay in vitro

Assays of cell migration were performed by using Transwell chambers (10 mm diameter, 8 µm pores, Costar Corp.) as described [27]. Capillary tube formation was performed as described on growth factor-reduced Matrigel [27].

### Confocal microscopy analysis

Microscopy was performed by using a Zeiss laser confocal microscope system (LSM 510) connected to an Axiovert 100M (Zeiss) and a Zeiss 63X objective. Images were acquired and loaded by using a TCS NT program (version 1.6.587). No further processing of the images was done except for changes in brightness/contrast to better visualize the data. Images were collected at 3.5 µm from the bottom and all conditions were imaged at the same distance from the glass.

### Calcium measurement assay

Changes in intracellular calcium were determined using the fluorescent calcium indicator Fluo-4 AM (Invitrogen). Cells were seeded on Lab-Tek chambered #1.0 borosilicate coverglass (LABTEK) for confocal live microscopy and serum starved overnight. Each well was then incubated with 200 µl of HBSS containing 0.5% BSA, 2 mM CaCl_2,_ 4 µM Fluo-4 for 45 min at 37°C. Cells were then washed twice with HBSS containing 0.5% BSA, 2 mM CaCl_2_ and incubated at 37°C in the same solution for 30 min to allow de-esterification of Fluo-4. Where indicated, 10 µM LY29400 was added after the first 15 minutes of the de-esterification passage for 15 min. Samples were analyzed using the Zeiss confocal microscope LSM 510 equipped with a chamber for live imaging at 37°C supplied with 5% CO_2_ using a 10X objective. After recording basal fluorescent, cells were stimulated with 100 ng/ml FGF-2 in the presence or in the absence of 10 µM LY294002 and fluorescence was measured for 10 min. At the end of each experiment, cells were stimulated with 1 mM ATP. ZEN Carl Zeiss software was used for acquisition and analysis of raw data. Fluorescence values of 40 cells per chamber were analyzed and expressed as mean of fluorescence.

## Results

### FGF-2 activates PLCγ1 in ECs

**Figure 1:**
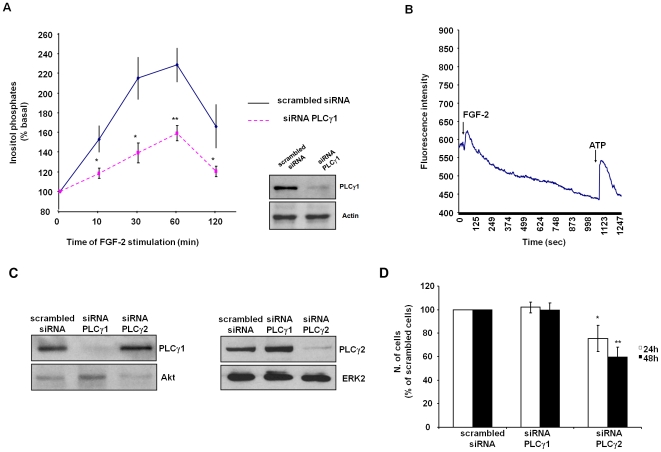
FGF-2 activates PLCγ1 in ECs. (A) HUVEC were transfected with scrambled siRNA or specific siRNA to downregulate PLCγ1 expression. After 24 h cells were labelled with [^3^H]myo inositol in M199 containing 2% FBS for a further 24 h and then stimulated with 100 ng/ml FGF-2 for the indicated times. Data indicate the total amount of inositol phosphates generated at the indicated times and are expressed as percentage of the basal inositol phosphates. Data are means±SEM of values obtained from 5 independent experiments. *p<0.05, **p<0.01. Blot shows PLCγ1 expression in lysates of HUVEC transfected with a control scrambled siRNA or siRNA targeting PLCγ1. Equal loading of proteins was assessed by using an anti actin antibody. (B) Intracellular calcium increase in serum starved HUVEC was determined as described in the Materials and Methods session. Cells were stimulated with 100 ng/ml FGF-2 and 1 mM ATP at the indicated times (arrows). Data are mean of values obtained from 4 independent experiments. (C) Expression levels of PLCγ1 or PLCγ2 in HUVEC transfected with the indicated siRNAs. Equal loading was confirmed using anti Akt or anti ERK antibodies. (D) HUVEC were transfected with scrambled siRNA or siRNAs targeting PLCγ1 or PLCγ2. The number of cells was determined by manual counting 24 h or 48 h after transfection. Data are expressed as percentage of cells transfected with scrambled siRNA at each time point and are means±SEM of values obtained from 6 independent experiments. *p<0.05, **p<0.01 vs HUVEC expressing siRNA PLCγ1. In C and D siRNA targeting PLCγ2 were from Dharmacon.

In order to determine whether PLCγ1 is a downstream target of FGF-2 in HUVEC we monitored the activation of the enzyme by metabolic labelling of cells and analysis of the total amount of inositol phosphates generated upon FGF-2 stimulation. Our data show that FGF-2 increased the levels of inositol phosphates in [^3^H]-labelled HUVEC (Figure 1A) and induced Ca^2+^ release from intracellular stores (Figure 1B), indicating activation of a PLC isoform. Downregulation of PLCγ1 expression using specific siRNA significantly reduced the FGF-2-dependent production of inositol phosphates, indicating that this isoform is specifically activated upon FGF-2 stimulation (Figure 1A). No reduction on the expression levels of the related PLC isoform PLCγ2 was observed in HUVEC transfected with the specific siRNA targeting PLCγ1 (Figure 1C). Interestingly, while PLCγ1 downregulation did not reduce the number of cells compared to HUVEC transfected with a control scrambled siRNA, expression of a specific siRNA targeting PLCγ2 strongly reduced the number of cells after 24 h or 48 h from transfection (Figure 1D). Similar results were obtained using a distinct siRNA targeting PLCγ2 (Figure S1). These data clearly indicate that PLCγ1 and PLCγ2 play distinct roles in HUVEC, with PLCγ2 but not PLCγ1 being possibly involved in proliferation and/or survival of cells.

### FGF-2-dependent PLCγ1 activation requires PI3K

**Figure 2:**
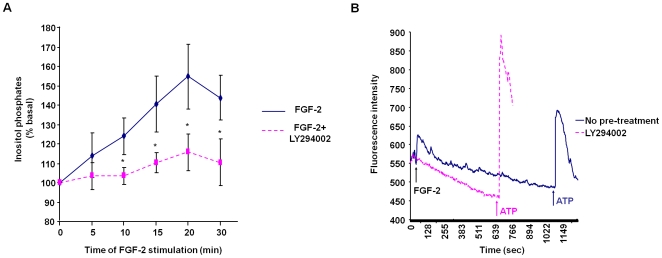
FGF-2 activates PLCγ1 in a mechanism involving PI3K activation. (A) HUVEC were labelled with [^3^H]myo inositol for 24 h and then pre-treated with 10 µM LY294002 for 15 min before stimulation with 100 ng/ml FGF-2. Data indicate the total amount of inositol phosphates generated at the indicated times and are expressed as percentages of the basal inositol phosphates. Data are means±SEM of values obtained from 9 independent experiments. *p<0.05. (B) Calcium release from serum starved HUVEC untreated or treated with 10 µM LY294002. Cells were stimulated with 100 ng/ml FGF-2 and 1 mM ATP at the indicated times (arrows). Data are mean of values obtained from 2 independent experiments.

In an effort to define the mechanism of FGF-2-induced PLCγ1 activation, we found that pre-treatment of cells with the reversible PI3K inhibitor LY294002 significantly inhibited the FGF-2-induced inositol phosphates production (Figure 2A). Similar results were obtained using the irreversible PI3K inhibitor wortmannin (Figure S2). Pre-treatment of cells with LY294002 completely blocked FGF-2-induced intracellular calcium increase without affecting the ATP-mediated release (Figure 2B). These data indicate that PI3K is required for full activation of PLCγ1, consistent with evidence indicating that PI3K can have a role in PLCγ1 activation [24], [28]. In addition we observed that the isolated N-terminal PH domain of PLCγ1 fused to green fluorescent protein (GFP-PH PLCγ1) translocated to the plasma membrane of HUVEC upon FGF-2 stimulation (Figure S3). Treatment of cells with wortmannin completely blocked this translocation (Figure S3), suggesting that binding of PLCγ1 PH domain to the FGF-2-dependent pool of PtdIns-3,4,5-P_3_ is responsible for translocation to the plasma membrane and subsequent activation of the enzyme. Taken together these data indicate that FGF-2 is able to activate PLCγ1 in ECs and that PI3K is required for full activation of the enzyme. In contrast the VEGF-dependent activation of PLCγ1 was not dependent on PI3K activity (Figure S4).

### FGF-2 activates PI3K in ECs

**Figure 3:**
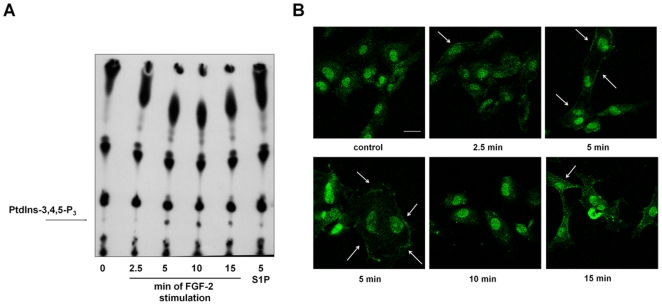
FGF-2 activates PI3K in HUVEC. (A) TLC analysis of phospholipids extracted from [^32^P]-labelled HUVEC stimulated with 100 ng/ml FGF-2 for the indicated times or 1 µM sphingosine-1-phosphate (S1P) for 5 min. Arrow indicates the PtdIns-3,4,5-P_3_ spot. (B) HUVEC grown on glass coverslips were incubated overnight in M199+0.5%FBS before stimulation with 100 ng/ml FGF-2 for the indicated times. Coverslips were then incubated with an anti PtdIns-3,4,5-P_3_ antibody and analyzed by confocal microscopy. Arrows indicate the plasma membrane localization of the phosphoinositide. Bar = 10 µm.

To analyze in greater detail FGF-2-mediated PI3K activation in HUVEC, we performed a time course analysis of PtdIns-3,4,5-P_3_ production *in vivo* by using TLC analysis of phospholipids extracted from [^32^P]-labelled HUVEC stimulated with FGF-2 for different times. The data indicate that FGF-2 generated PtdIns-3,4,5-P_3_ with a peak at 5 and 10 min of stimulation (Figure 3A). Confocal microscopy analysis using a specific anti PtdIns-3,4,5-P_3_ antibody confirmed the FGF-2-induced production of this phosphoinositide at the plasma membrane of HUVEC (Figure 3B). These data indicate that FGF-2 rapidly and transiently activates PI3K in HUVEC.

### PLCγ1 is necessary for FGF-2-dependent migration and capillary tubule formation by ECs

**Figure 4:**
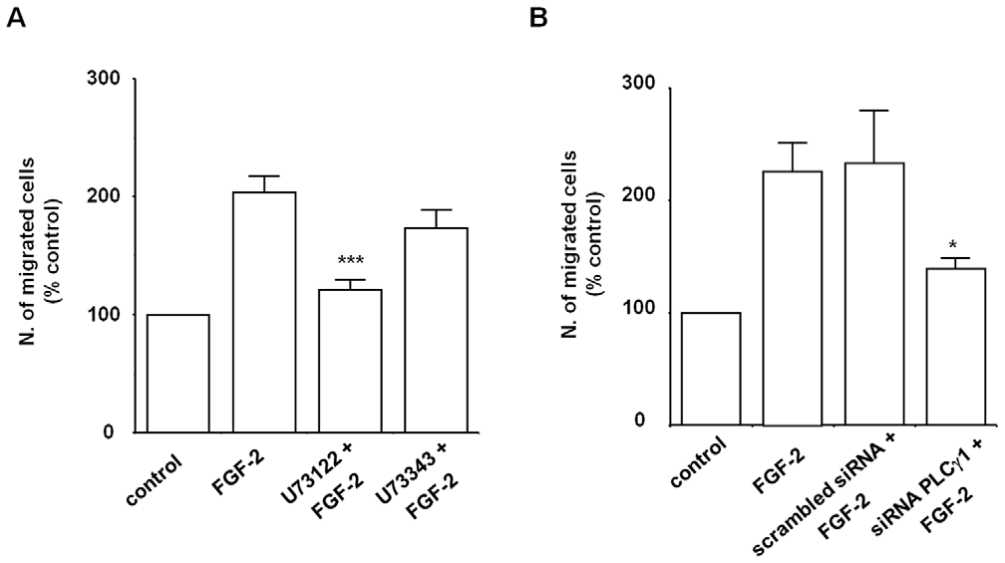
PLCγ1 is required for FGF-2-induced cell migration. (A) HUVEC were incubated overnight in M199+0.5%FBS before treatment with 5 µM U73122 or U73343 for 30 min. Migration of cells in the presence of 100 ng/ml FGF-2 was assessed by Transwell assays. Data are means±SEM of values obtained from 7 independent experiments and are expressed as percentage of cells migrating in the absence of FGF-2 (control) ***p<0.001. (B) HUVEC were transfected with scrambled siRNA or siRNA to downregulate PLCγ1 expression. After 24 h cells were incubated overnight in M199+0.5% FBS. Migration of cells in the presence of 100 ng/ml FGF-2 was then assessed by Transwell assays. Data are means±SEM of values obtained from 7-10 independent experiments *p<0.05.

The results so far indicated that FGF-2 can activate PLCγ1 in HUVEC and that PI3K activation is required for full activation of the enzyme. Since we have reported that the PI3K-dependent activation of PLCγ1 is a key event in migration of breast cancer cells [29] and PLCγ1 is necessary for breast cancer migration and invasion [30], we investigated whether this novel PI3K/PLCγ1 pathway was involved in the FGF-2-dependent migration of HUVEC. We have already reported that PI3K is required in such process [27]. To test the role of PLCγ1 we first examined the effect of the PLC inhibitor U73122 on cell migration. Transwell assays revealed that blockade of PLC activity completely abrogated the FGF-2-induced migration in HUVEC whereas no significant effect was observed when cells were treated with the inactive structural analogue U73343 (Figure 4A). Moreover, downregulation of PLCγ1 using siRNA specifically inhibited FGF-2-dependent migration of HUVEC (Figure 4B). These data indicate that the PI3K/PLCγ1 pathway is involved in FGF-2-induced cell migration.

**Figure 5:**
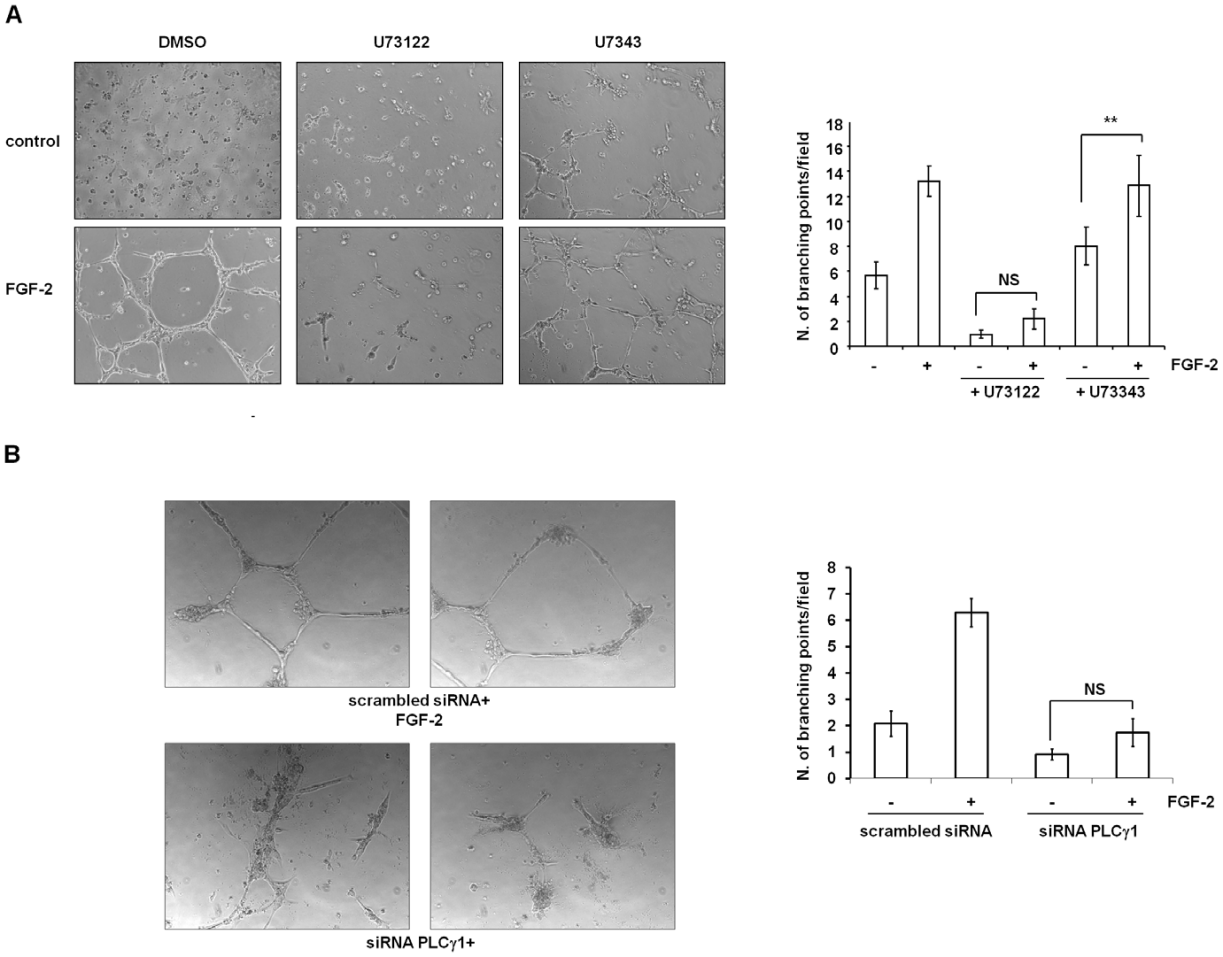
PLCγ1 is necessary for FGF-2-induced tubulogenesis *in vitro*. (A) Representative images of HUVEC on Matrigel in the absence or presence of FGF-2 plus 2 µM of the PLC inhibitor U73122 or its inactive analogue U73343. Plot represents a quantitative analysis of the experiments obtained by counting the number of branch points from five fields. Data are means±SEM of values obtained from 12 independent experiments.**p<0.01. NS = not significant. (B) Representative images of HUVEC transfected with the scrambled siRNA or siRNA targeting PLCγ1 on Matrigel in the presence of FGF-2. Quantitative analysis show data as means±SEM of values obtained from 5 independent experiments. NS = not significant.

Since migration is a key step in angiogenesis, we then tested whether PLCγ1 was involved in this process by performing *in vitro* angiogenesis assays. Capillary tubule formation assay on Matrigel revealed that treatment of HUVEC with the PLC inhibitor U73122 completely blocked the formation of three-dimensional structures whereas U73343 had no effect (Figure 5A). Similar results were obtained upon PLCγ1 downregulation in HUVEC (Figure 5B). These data indicate that PLCγ1 plays a key role in FGF-2-dependent EC remodelling and angiogenesis, most likely by regulating cell migration.

### PLCγ1 mediates Akt phosphorylation through PKCα activation

**Figure 6:**
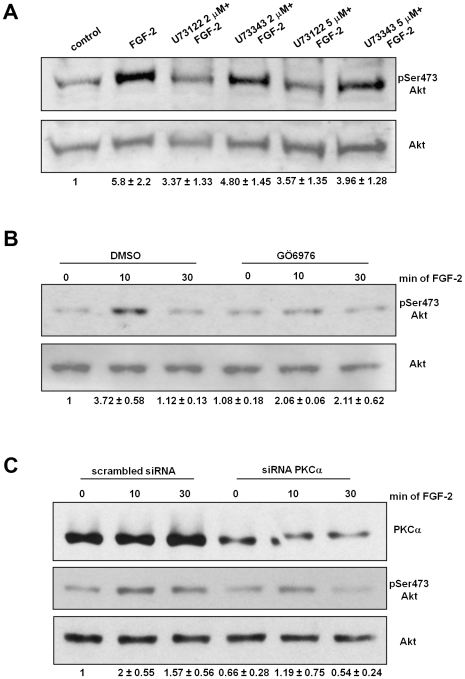
PLCγ1-dependent PKCα activation mediates FGF-2-induced Akt activation. HUVEC were incubated overnight in M199+0.5%FBS and then treated with the indicated concentrations of U73122 or U73343 for 30 min (A) or 10 µM of the conventional PKCs inhibitor Gö6976 (B) before stimulation with 100 ng/ml FGF-2 for 10 min. (C) HUVEC were transfected with scrambled siRNA or siRNAs targeting PKCα. After 24 h cells were serum deprived overnight and then stimulated with 100 ng/ml FGF-2 for the indicated times. Phosphorylation of Akt at Ser473 was assessed by using a specific antibody. Membranes were then stripped and re-probed with an anti Akt. Blots are representative of 7 (A) and 3 (B,C) independent experiments. In all cases, quantitative analysis of the band intensities was performed. Data represent values of pSer473 Akt/Akt expressed as fold increase over control.

We recently demonstrated that FGF-2-dependent migration and tubulogenesis requires activation of the PI3K target PKB/Akt [27]. The observation that blockade of PLCγ1 was able to inhibit both processes prompted us to investigate whether a PLCγ1-dependent mechanism was involved in Akt activation. This hypothesis was further supported by recent data demonstrating that PKCα is involved in Akt activation in ECs [31], [32]. Since PLCγ1 mediates PKCα activation, we investigated whether PLCγ1-mediated PKCα activation was required for FGF-2-dependent Akt activation. Blockade of PLC activity inhibited FGF-2-induced phosphorylation of Akt at residue Ser473 in HUVEC (Figure 6A). No effect was observed with the inactive analogue U73343 (Figure 6A). Furthermore we observed that treatment of cells with the conventional PKC inhibitor Gö6976 (Figure 6B) and specific PKCα knock down (Figure 6C) markedly inhibited the FGF-2-mediated Akt phosphorylation, indicating that a PLCγ1-mediated PKCα activation is necessary for FGF-2-mediated Akt activation. Taken together these data indicate that FGF-2-induced, PI3K-mediated Akt phosphorylation at residue Ser473 depends on PLCγ1 activation and subsequent activation of PKCα.

### PKCα is required for FGF-2-dependent migration

**Figure 7:**
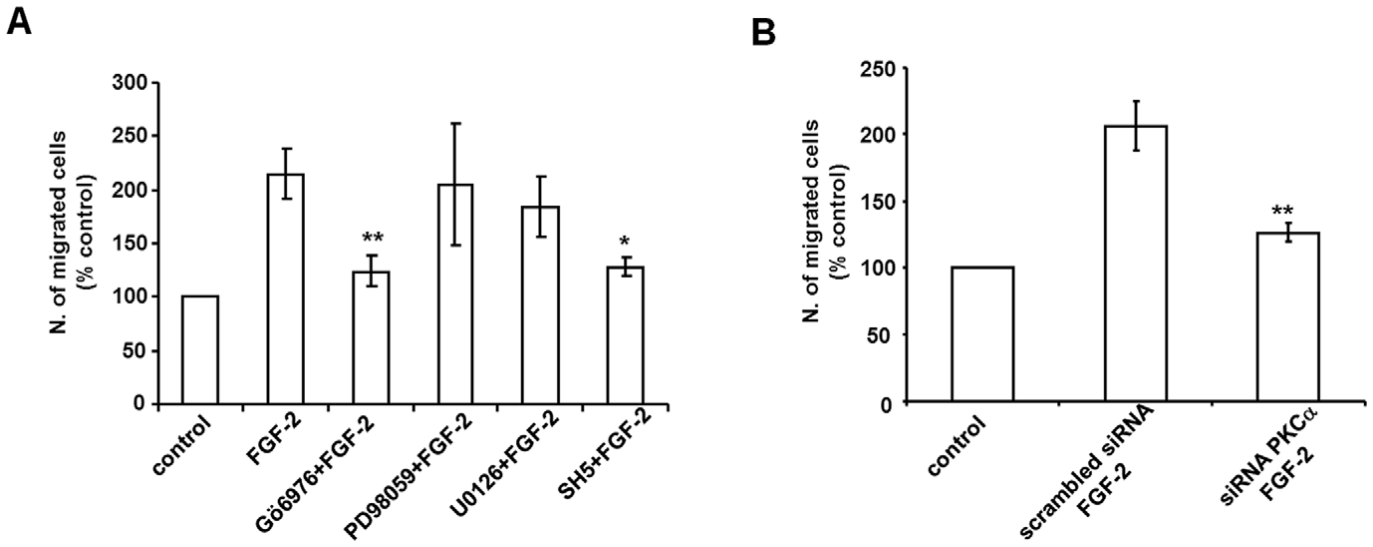
PKCα is required for FGF-2-induced cell migration. (A) HUVEC were incubated overnight in M199+0.5%FBS before treatment with 2 µM Gö6796, 50 µM or 10 µM of the MEK inhibitors PD98059 and U0126 respectively or 50 µM of the Akt inhibitor SH5 for 30 min. Migration of cells in the presence of 100 ng/ml FGF-2 was then assessed by Transwell assays. Data are means±SEM of values obtained from 2-7 independent experiments *p<0.05, **p<0.01. (B) HUVEC were transfected with scrambled siRNA or siRNA to downregulate PKCα expression. After 24 h cells were incubated overnight in M199+0.5% FBS. Migration of cells in the presence of 100 ng/ml FGF-2 was then assessed by Transwell assays. Data are means±SEM of values obtained from 5 independent experiments **p<0.01.

We next investigated the role of PKCα in Akt-dependent cellular functions. In particular, since we observed that both PLCγ1 and Akt are required for FGF-2-dependent migration, we investigated whether PKCα was also involved in this process. Transwell assays revealed that PKC inhibition with Gö6976 (Figure 7A) completely blocked FGF-2-dependent migration of HUVEC. Consistent with our previous work demonstrating the role of Akt in FGF-2-induced migration of HUVEC [27] we found that the Akt inhibitor SH5 strongly impaired cell migration (Figure 7A) whereas no effect was observed using MEK inhibitors (Figure 7A). Specific PKCα knock down also resulted in complete inhibition of FGF-2-mediated cells migration (Figure 7B). These data indicate that the novel PI3K/PLCγ1/PKCα/Akt pathway is involved in FGF-2-stimulated EC migration.

## Discussion

Here we show that PLCγ1 is activated upon FGF-2 stimulation in HUVEC and that upstream activation of PI3K is required for full activation of the enzyme. PLCγ1 activation is necessary for FGF-2-induced capillary tube formation *in vitro*. This process is the result of distinct intracellular events (cell proliferation, apoptosis, migration) and our data indicate that the role of PLCγ1 in tubulogenesis is associated with its role in cell migration.

The mechanisms of FGF-dependent activation of PI3K and PLCγ1 have been controversial. Early studies showed that p85, the regulatory subunit of PI3K, did not coimmunoprecipitate with FGFR1 and no PI3K activity was detected in anti FGFR1 immunoprecipitates [33]. However, subsequent work revealed that FGF-2 increases PI3K activity in anti-p85 or anti-p110 immunoprecipitates [34] and that assembly of a multi-docking protein complex is necessary for the FGF-2-mediated activation of PI3K [17]. By directly monitoring generation of its lipid product PtdIns-3,4,5-P_3_, we demonstrate here that FGF-2 activates PI3K in HUVECs and that this enzyme is involved in PLCγ1 activation. Although FGF-induced PLCγ1 phosphorylation and activation were previously observed and characterised in fibroblasts and in cell lines overexpressing FGFR1 [35], other studies reported that FGF was not able to induce PLCγ1 tyrosine phosphorylation and activation in choroidal ECs [36]. Similarly, no tyrosine phosphorylation of PLCγ1 was detected in FGF-stimulated HUVEC [37], although total inositol phosphates production was not monitored in this study. Here we demonstrate that FGF-2 is indeed able to stimulate PLC activity. Moreover selective downregulation of PLCγ1 reveals the specific activation of this isoform upon FGF-2 stimulation, in agreement with the reported FGF-2-dependent PLCγ1 activation in corneal ECs [38]. Although the time course of stimulation performed in the previous report was different compared to our study, it is interesting to notice that the fold increase of total inositol phosphates accumulation upon 1 h of FGF-2 stimulation is very similar between the two studies. We further show that downregulation of PLCγ1 does not affect the expression levels of the related PLC isoform PLCγ2. Interestingly, downregulation of PLCγ2 but not PLCγ1 specifically reduces the number of cells compared to HUVECs expressing a scrambled siRNA, suggesting a potential role for PLCγ2 in proliferation and/or survival of cells. These results are consistent with accumulating evidence revealing distinct, not overlapping functions for PLCγ1 and PLCγ2 [39]–[42].

Our study indicates that PI3K activation is required for PLCγ1 activation, consistent with our previous work describing the interaction of PLCγ1 PH domain with the PI3K product PtdIns-3,4,5-P_3_ and its role in membrane targeting of the enzyme [24] and with evidence indicating that tyrosine phosphorylation is not sufficient for PLCγ1 activity [28], [43], [44]. Our data clearly indicate that monitoring PLCγ1 activation only by analysing its tyrosine phosphorylation does not take into account the possibility of additional and/or alternative mechanisms of activation of the enzyme [28]. In this respect it must be observed that, although no activation of PLCγ1 was detected in porcine aortic ECs overexpressing a mutant FGFR1 unable to induce tyrosine phosphorylation of PLCγ1 (FGFR1 Y766F) [45], a detailed analysis of PLCγ1 activation at different times of stimulation was not performed in this report. Indeed by performing accurate time course experiments we observed that at specific time points a PI3K-dependent PLC activity can be detected in cell lines overexpressing the FGFR1 Y766F mutant (data not shown). It must be noted that PI3K is involved in FGF-2-dependent but not in VEGF-dependent PLCγ1 activation suggesting that different mechanisms of PLCγ1 activation exist in different cellular contexts.

We further show that the PI3K-dependent PLCγ1 activation is required for FGF-2-dependent migration of HUVEC. Previous data indicated that PLCγ1 was not required for migration downstream of FGFR1 activation [45] but it must be noted that these data were obtained in cells overexpressing the FGFR1 Y766F mutant therefore in the absence of PLCγ1 tyrosine phosphorylation. Since these cells were still able to migrate in response to FGFR1 activation it was concluded that PLCγ1 had no role in this process [45]. However, as discussed above, such a conclusion did not take into account the possibility of the PI3K-mediated activation of PLCγ1. The role of PLCγ1 in EC migration is consistent with the reported role of this enzyme in cell migration, invasion and spreading of other cell types [46]–[49]. In particular we demonstrated that PI3K-mediated PLCγ1 activation is required for epidermal growth factor-induced migration of breast cancer cells [29]. More recently, we reported that PLCγ1 is required for breast cancer cells migration and invasion [30]. Our present and previous data indicate that both PI3K [27] and PLCγ1 are required for FGF-2-induced tubulogenesis *in vitro*. Recent data have suggested that PI3K and PLCγ1 have opposite effects on capillary tube formation *in vitro* with PI3K promoting tubule formation and PLCγ1 regulating tubule regression [50]. The apparent discrepancies between this report and our present data are likely due to the different experimental conditions. Furthermore, our results are in agreement with data demonstrating the key role of PLCγ1 in HUVEC cell elongation and network formation on Matrigel [49]. Our data reveal that the FGF-2-induced tubule formation is also dependent on PLCγ1 activation. It must be pointed out that we cannot completely rule out the possibility that in our experimental conditions the detected tubule formation is not directly dependent on FGF-2 stimulation. The possibility exists that FGF-2 stimulation of HUVEC upon overnight serum starvation induces survival of cells which are then able to migrate in response to extracellular matrix components, as previously reported [49]. Nevertheless our data indicate the key role of PLCγ1 in this process, suggesting that activation of a PI3K/PLCγ1 pathway is involved in FGF-2-dependent angiogenesis. This would be consistent with recent data in literature. Indeed it has been reported that y10 (the zebra fish homologue of PLCγ1) mutant embryos display specific defect in the formation of arteries, but not veins [23]. Sequence analysis revealed that the majority of the mutations in y10 mutants occur in the PH domain [23] and in particular in the β3/β4 region of the PH domain that we demonstrated to be important for PtdIns-3,4,5-P_3_ binding and growth factor-induced membrane localization of PLCγ1 [24]. These data suggest that a PI3K-dependent regulation of PLCγ1 might be involved in vasculogenesis.

In an effort to define the mechanism of PLCγ1-dependent regulation of EC tubulogenesis we found that activation of a PLCγ1/PKCα pathway is necessary for FGF-2-induced Akt phosphorylation. Akt is involved in physiological and pathological angiogenesis through effects in both EC and cells producing endothelial signals such as tumour cells [51]-[55]. Furthermore Akt is required for EC migration [56] and we reported that this enzyme is necessary for both FGF-2-induced migration and tubulogenesis in HUVEC [27]. Akt activation requires its phosphorylation at Thr308 by phosphoinositide-dependent kinase-1 [57], [58] and at Ser473. Although the mTORC2 complex, containing the enzyme mammalian target of rapamycin, has been shown to mediate Ser473 phosphorylation in several cell types [59], the precise mechanism of Akt Ser473 phosphorylation and the link between PI3K and this crucial event in Akt activation remains unclear. Our data here reveal a novel and critical role for a PLCγ1/PKCα pathway in mediating FGF stimulation of Akt phosphorylation at Ser473. This data is consistent with previous reports indicating a role for PKC in Akt phosphorylation in ECs [31], [32]. Interestingly it was recently reported that a PLCγ1-dependent PKC activation is necessary for Akt phosphorylation at Ser473 in monocytes stimulated with phospholipase A_2_-modified low density lipoprotein [60]. On the other hand, we did not detect impairment of Ser473 Akt phosphorylation in breast cancer cells stimulated with epidermal growth factor upon downregulation of PLCγ1 [30] suggesting that the role of PLCγ1 in mediating Akt phosphorylation may be cell and stimulus specific. Due to the variety of intracellular events requiring Akt activation it is likely that identification of PLCγ1 as a key mediator in its activation will lead to the discovery of novel roles for PLCγ1 in different intracellular functions.

EC lining the lumen of all blood vessels play essential roles in maintaining normal vascular function and in vascular homeostasis [1]. EC migration is a crucial step in angiogenesis and is essential for regeneration of endothelia damaged by balloon catheter angioplasty and stent placement. Furthermore EC migration is a prerequisite for the formation of collateral blood vessels in ischaemic heart and peripheral vascular disease [1]. A better understanding of the factors and intracellular processes governing EC migration will have profound implications not only for cardiovascular disease but also for a better understanding of embryogenesis and reproduction. Furthermore due to the emerging role of FGF-2 in tumour angiogenesis and its potential role in drug resistance to anti-angiogenic agents, understanding the FGF-dependent signalling pathways will prove useful to design novel potential strategies of intervention. In this respect our present data, together with our previous work indicating the critical role of PLCγ1 in metastasis development and progression [30] suggest that targeting PLCγ1 may represent a useful strategy to inhibit metastasis growth and possibly tumour angiogenesis simultaneously. In this respect identification of PLCγ1 as a critical player in regulation of FGF-induced EC migration and tubulogenesis and in modulating Akt activation will have both biological and potential therapeutic implications.

## Supporting Information

Figure S1 Effects of downregulation of PLCγ1 and PLCγ2 on HUVEC. (A) Expression levels of PLCγ1 or PLCγ2 in HUVEC transfected with the indicated siRNAs. Equal loading was confirmed using anti Akt or anti ERK antibodies. (B) HUVEC were transfected with scrambled siRNA or siRNAs targeting PLCγ1 or PLCγ2. After 24 h, cells were starved overnight in M199+0.5% FBS and then the number of cells was determined by manual counting. Data are expressed as percentage of cells transfected with scrambled siRNA and are means±SEM of values obtained from 5 independent experiments. *p<0.05, **p<0.01 vs HUVEC expressing siRNA PLCγ1. In these experiments siRNA targeting PLCγ2 was from Qiagen.
(1.56 MB TIF)

Figure S2 FGF-2 activates PLCγ1 in a mechanism involving PI3K activation. HUVEC were labelled with [3H]myo inositol for 24 h and then pre-treated with 100 nM wortmannin for 15 min before stimulation with 100 ng/ml FGF-2. Data indicate the total amount of inositol phosphates generated at the indicated times and are expressed as percentages of the basal inositol phosphates. Data are means±SEM of values obtained from 1–2 independent experiments.
(3.73 MB TIF)

Figure S3 PI3K regulates FGF-2-induced PLCγ1 recruitment to the plasma membrane. HUVEC were transfected with a GFP-tagged PLCγ1 PH domain and plated on glass coverslips. After 24 h cells were serum deprived overnight in M199+0.5% FBS and then left untreated or pre-treated with 100 nM wortmannin for 15 min before stimulation with 100 ng/ml FGF-2 for 10 min. Coverslips were fixed and analyzed by confocal microscopy.
(6.24 MB TIF)

Figure S4 VEGF-mediated activation of PLCγ1 is not dependent on PI3K activation. HUVEC were labelled with [3H]myo inositol for 24 h and then pre-treated with 10 µM LY294002 (A) or 100 nM wortmannin (B) for 15 min before stimulation with 10 ng/ml VEGF. Data indicate the total amount of inositol phosphates generated at the indicated times and are expressed as percentages of the basal inositol phosphates. Data are means±SEM of values obtained from 1–3 (A) or 1–4 independent experiments (B).
(6.07 MB TIF)
