# Supplementary figures and images for: A Phosphoinositide 3-Kinase/Phospholipase Cgamma1 Pathway Regulates Fibroblast Growth Factor-Induced Capillary Tube Formation
Source: PLoS One. 2009 Dec 14;4(12):e8285. doi: 10.1371/journal.pone.0008285 (PMC2788267; doi:10.1371/journal.pone.0008285)

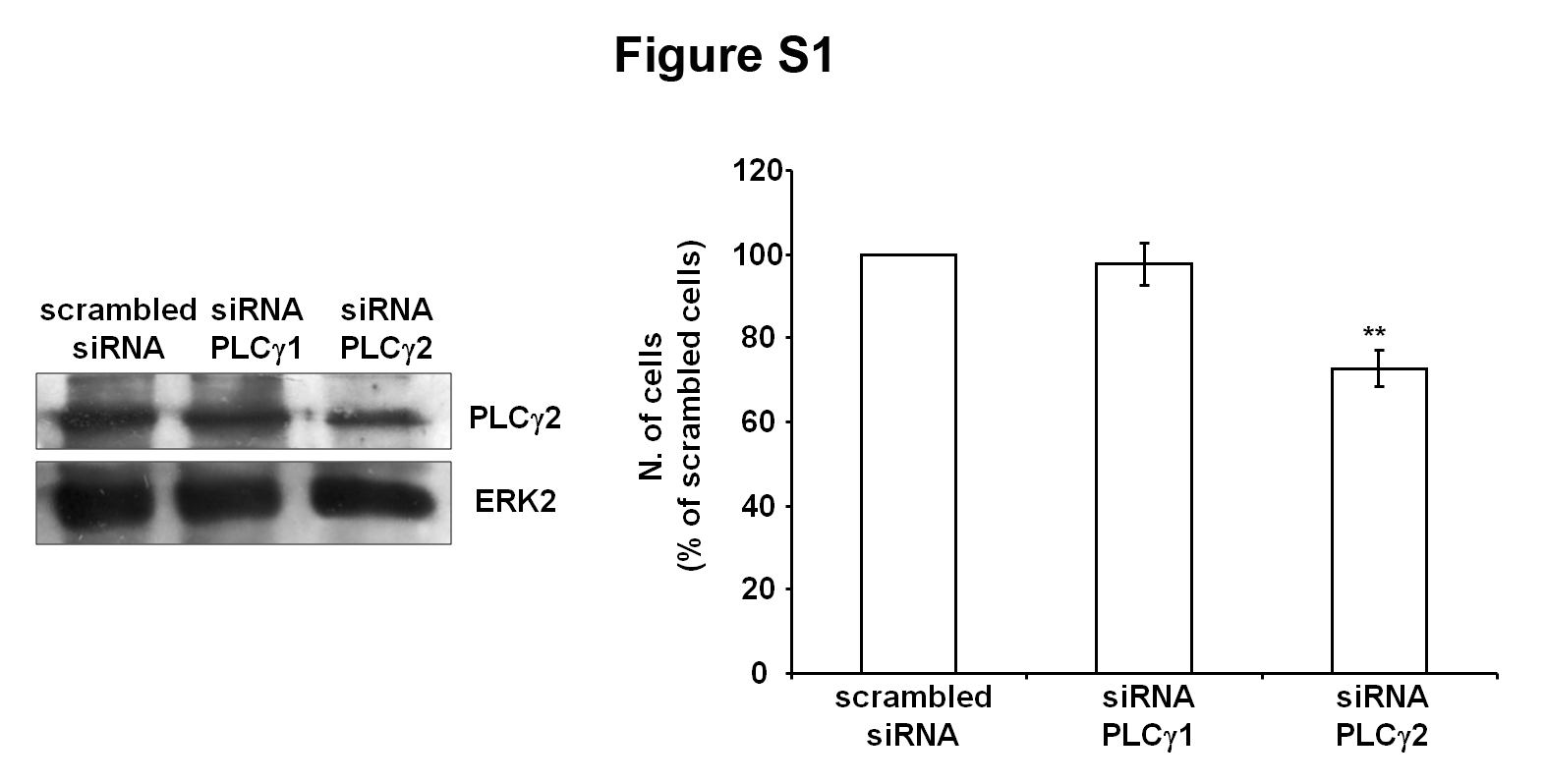

Supplement: Figure S1 — Effects of downregulation of PLCγ1 and PLCγ2 on HUVEC. (A) Expression levels of PLCγ1 or PLCγ2 in HUVEC transfected with the indicated siRNAs. Equal loading was confirmed using anti Akt or anti ERK antibodies. (B) HUVEC were transfected with scrambled siRNA or siRNAs targeting PLCγ1 or PLCγ2. After 24 h, cells were starved overnight in M199+0.5% FBS and then the number of cells was determined by manual counting. Data are expressed as percentage of cells transfected with scrambled siRNA and are means±SEM of values obtained from 5 independent experiments. *p<0.05, **p<0.01 vs HUVEC expressing siRNA PLCγ1. In these experiments siRNA targeting PLCγ2 was from Qiagen. (1.56 MB TIF) [file pone.0008285.s001.tif]

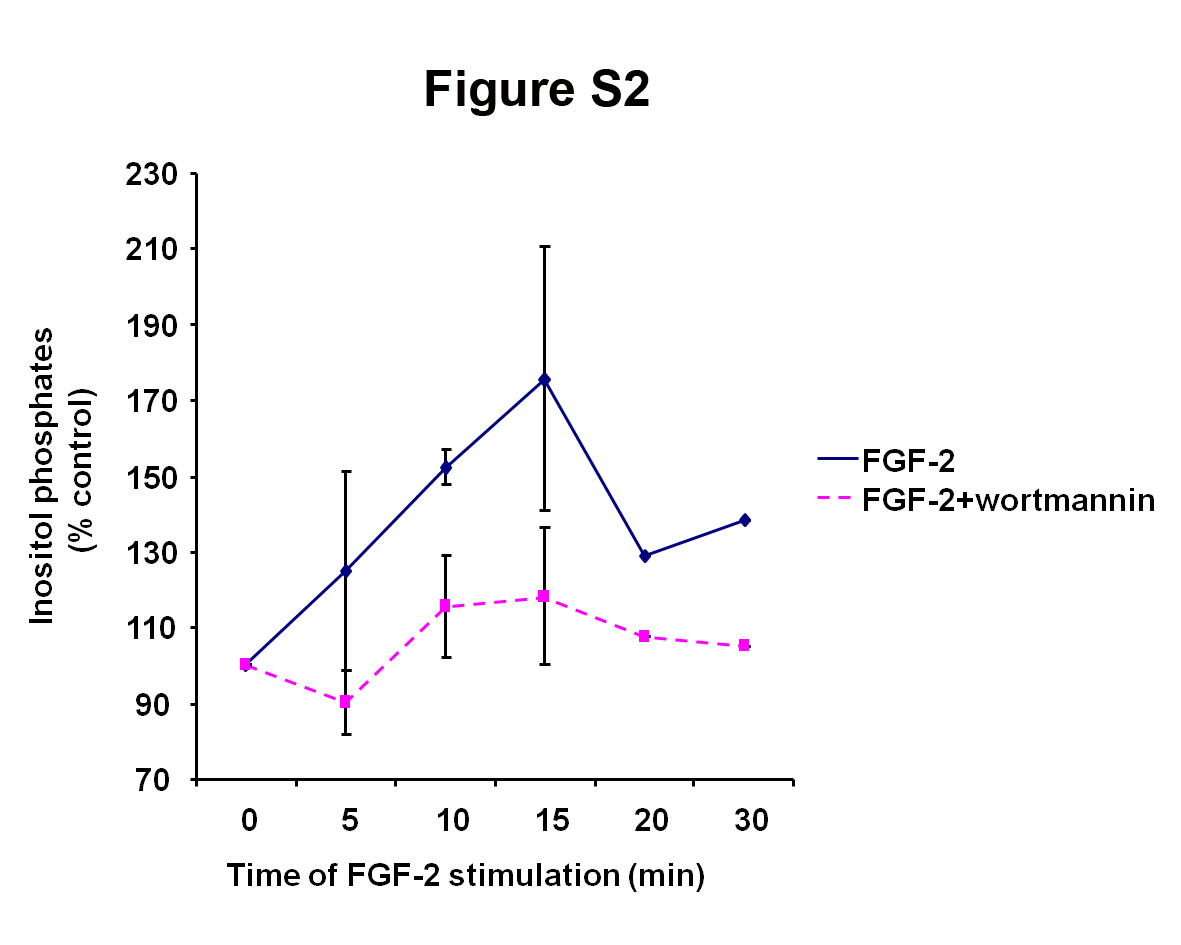

Supplement: Figure S2 — FGF-2 activates PLCγ1 in a mechanism involving PI3K activation. HUVEC were labelled with [3H]myo inositol for 24 h and then pre-treated with 100 nM wortmannin for 15 min before stimulation with 100 ng/ml FGF-2. Data indicate the total amount of inositol phosphates generated at the indicated times and are expressed as percentages of the basal inositol phosphates. Data are means±SEM of values obtained from 1–2 independent experiments. (3.73 MB TIF) [file pone.0008285.s002.tif]

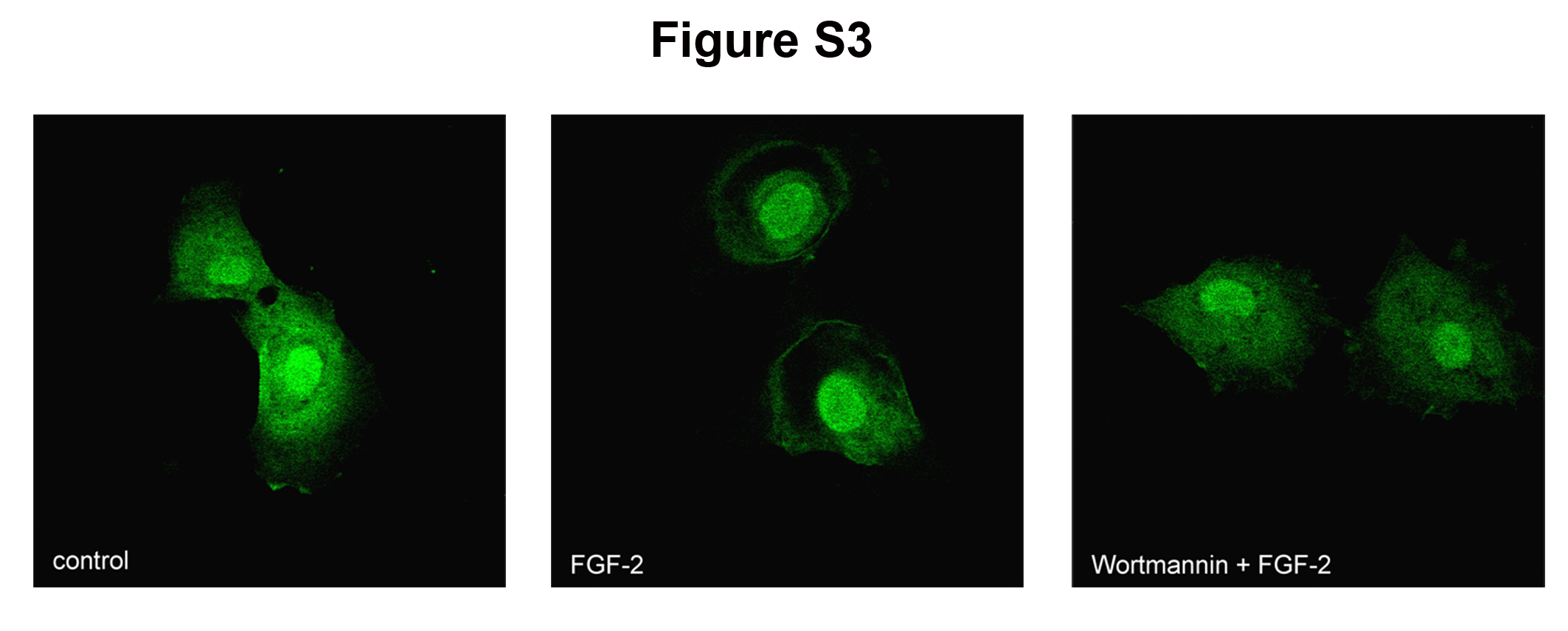

Supplement: Figure S3 — PI3K regulates FGF-2-induced PLCγ1 recruitment to the plasma membrane. HUVEC were transfected with a GFP-tagged PLCγ1 PH domain and plated on glass coverslips. After 24 h cells were serum deprived overnight in M199+0.5% FBS and then left untreated or pre-treated with 100 nM wortmannin for 15 min before stimulation with 100 ng/ml FGF-2 for 10 min. Coverslips were fixed and analyzed by confocal microscopy. (6.24 MB TIF) [file pone.0008285.s003.tif]

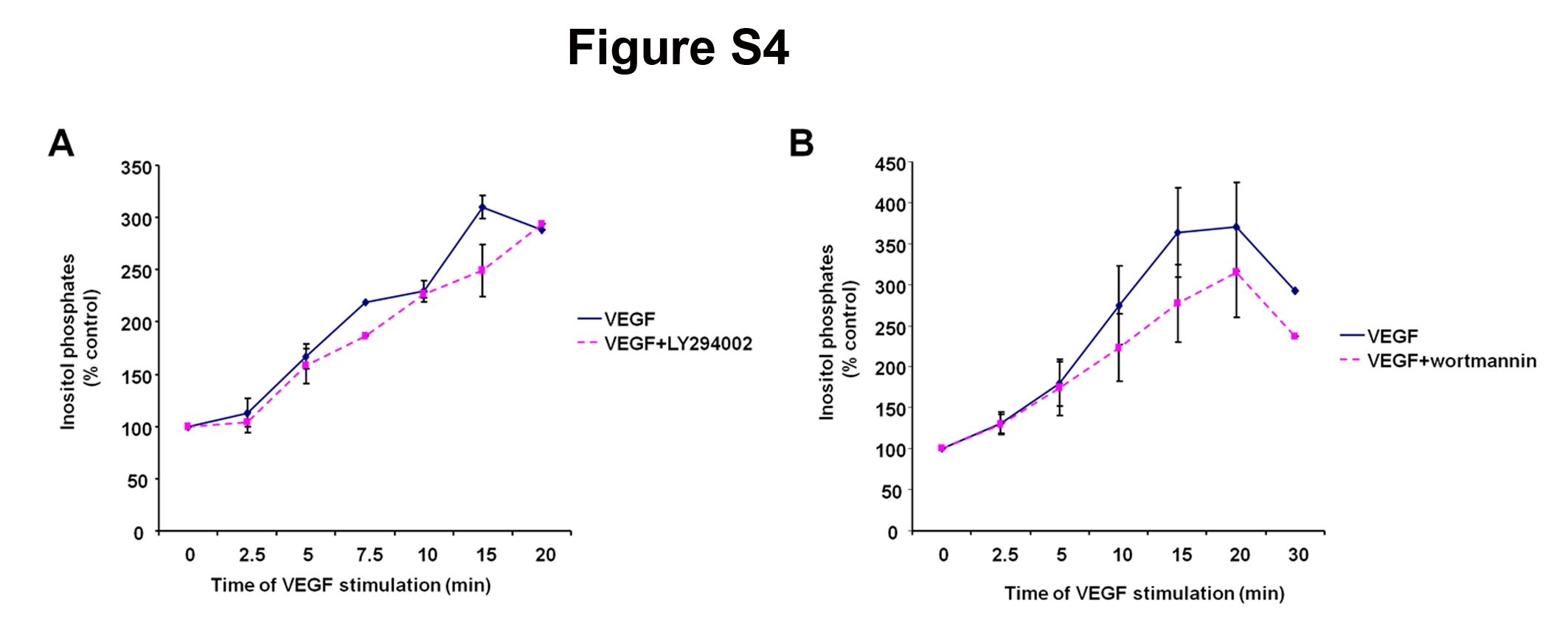

Supplement: Figure S4 — VEGF-mediated activation of PLCγ1 is not dependent on PI3K activation. HUVEC were labelled with [3H]myo inositol for 24 h and then pre-treated with 10 µM LY294002 (A) or 100 nM wortmannin (B) for 15 min before stimulation with 10 ng/ml VEGF. Data indicate the total amount of inositol phosphates generated at the indicated times and are expressed as percentages of the basal inositol phosphates. Data are means±SEM of values obtained from 1–3 (A) or 1–4 independent experiments (B). (6.07 MB TIF) [file pone.0008285.s004.tif]
